# Low Levels of Fruit Nitrogen as Drivers for the Evolution of Madagascar’s Primate Communities

**DOI:** 10.1038/s41598-017-13906-y

**Published:** 2017-10-31

**Authors:** Giuseppe Donati, Luca Santini, Timothy M. Eppley, Summer J. Arrigo-Nelson, Michela Balestri, Sue Boinski, An Bollen, LeAndra L. Bridgeman, Marco Campera, Valentina Carrai, Mukesh K. Chalise, Abigail Derby Lewis, Gottfried Hohmann, Margaret F. Kinnaird, Andreas Koenig, Martin Kowalewski, Petra Lahann, Matthew R. McLennan, Anna K. I. Nekaris, Vincent Nijman, Ivan Norscia, Julia Ostner, Sandra Y. Polowinsky, Oliver Schülke, Christoph Schwitzer, Pablo R. Stevenson, Mauricio G. Talebi, Chia Tan, Irene Tomaschewski, Erin R. Vogel, Patricia C. Wright, Jörg U. Ganzhorn

**Affiliations:** 1 Department of Social Sciences, Oxford Brookes University, Oxford, OX3 0BP UK; 2 Department of Environmental Science, Institute for Wetland and Water Research, Faculty of Science, Radboud University, P.O. Box 9010, NL-6500 GL Nijmegen, The Netherlands; 3 Department of Biological and Environmental Sciences, California University of Pennsylvania, 250 University Ave – Box 45, California, PA 15419 USA; 4 Department of Anthropology, University of Florida, Gainesville, FL USA; 5 Prospect Consulting and Services, Rue de Prince Royal 83, 1050 Brussels, Belgium; 6 Department of Anthropology, Washington University, St Louis, MO USA; 7 Biology Department, University of Pisa, Via A. Volta, 4, I-56126 Pisa, Italy; 8 Central Department of Zoology, Tribhuvan University, Kirtipur, Kathmandu, Nepal; 9 Science Action Center, The Field Museum, 1400 S. Lake Shore Drive, Chicago, IL 60605 USA; 10 Biozentrum Grindel, Dept. Animal Ecology and Conservation, Martin-Luther-King Platz 3, Universität Hamburg, 20146 Hamburg, Germany; 11 Max Planck Institute for Evolutionary Anthropology, Department of Primatology, Deutscher Platz 6, D-04103 Leipzig, Germany; 12 Mpala Research Centre, PO Box 555 Nanyuki, Kenya; 13 Department of Anthropology and Interdepartmental Doctoral Program in Anthropological Sciences, Stony Brook University, Stony Brook, NY 11794-4364 USA; 14 Estación Biológica Corrientes (Museo Argentino de Cs. Naturales)-CONICET, Corrientes, Argentina; 15 Museo di Storia Naturale, University of Pisa, Via Roma, 79, 56011 Calci, PI Italy; 16 Research Group Primate Social Evolution, German Primate Center, Kellnerweg 4, 37077 Göttingen, Germany; 17 Bristol Zoological Society, Clifton, Bristol, BS8 3HA UK; 18 Departamento de Ciencias Biológicas, Universidad de Los Andes, Cr. 1 no, 18A-10 Bogotá, D.C. Colombia; 19 Departamento de Ciências Ambientais/Programa de Pós Graduação Análise Ambiental Integrada, Universidade Federal de São Paulo, Campus Diadema SP, Brazil; 20 San Diego Zoo Institute for Conservation Research, 15600 San Pasqual Valley Road, Escondido, CA 92027-7000 USA; 21 Department of Anthropology and Center for Human Evolutionary Studies, Rutgers University, 131 George Street, New Brunswick, NJ 08901-1414 USA; 22 San Diego Zoo Global, Institute for Conservation Research, PO Box 120551, San Diego, CA USA

## Abstract

The uneven representation of frugivorous mammals and birds across tropical regions – high in the New World, low in Madagascar and intermediate in Africa and Asia – represents a long-standing enigma in ecology. Several hypotheses have been proposed to explain these differences but the ultimate drivers remain unclear. Here, we tested the hypothesis that fruits in Madagascar contain insufficient nitrogen to meet primate metabolic requirements, thus constraining the evolution of frugivory. We performed a global analysis of nitrogen in fruits consumed by primates, as collated from 79 studies. Our results showed that average frugivory among lemur communities was lower compared to New World and Asian-African primate communities. Fruits in Madagascar contain lower average nitrogen than those in the New World and Old World. Nitrogen content in the overall diets of primate species did not differ significantly between major taxonomic radiations. There is no relationship between fruit protein and the degree of frugivory among primates either globally or within regions, with the exception of Madagascar. This suggests that low protein availability in fruits influences current lemur communities to select for protein from other sources, whereas in the New World and Old World other factors are more significant in shaping primate communities.

## Introduction

The evolution of plant-frugivore interactions is considered to have contributed to the high diversity of tropical mammals and birds^1,2^. This diversity is the consequence of a long evolutionary history where the radiation of angiosperms triggered the emergence of frugivorous vertebrates^2^, which in turn are essential for the dispersal of most tropical plant species^3,4^. Among vertebrates, however, strict frugivory is rare since fruits are distributed patchily in space and time^5^ and, although easy to digest, are assumed to contain too little proteins to meet species’ metabolic requirements^6,7^. Nitrogen, the fundamental component of proteins, has been suggested as a limiting factor for the survival of vertebrate communities in general^8–10^. Thus, most frugivorous mammals and birds supplement their diets with protein-rich foods, such as leaves or invertebrates, to satisfy their nitrogen needs^3,11^.

Due to the key role of proteins, primate feeding guilds, within which frugivory is widely represented, are hypothesized to have evolved under the constraints of nitrogen availability^12^. For example, among folivorous primates the ratio between protein and fiber has been found to be a powerful predictor of food choice and population densities^13–19^, but see^20,21^. While some primates appear to maximize protein intake^22,23^, others apparently do not^24,25^. At least for folivorous species, the strength of this selection depends on the protein availability within the environment^26^. Maintaining a balanced protein intake is particularly challenging for frugivorous primates, since they lack physiological adaptations to optimize nitrogen extraction from food and thus must increase their total food intake in order to meet their requirements^7,27^. Recent investigations based on fine-grained analyses of nutrient intake suggest that although protein maximization does not explain spider monkey (*Ateles chamek*) feeding choices, this frugivorous species maintains a relatively constant protein intake regardless of season, sex, or available food^10^.

Frugivory seems to have evolved independently numerous times in primates^28^. The New World has a greater number of frugivorous families and species compared with the Old World radiations of Africa and Asia, while Madagascar appears to have a paucity of taxa in this dietary guild^29,30^. Recent data indicate that when food intake, rather than feeding time, is used to quantify diet, even the most folivorous of New World primates include a considerable proportion of fruits in their diet^31^. Unlike the New World, many Malagasy primates are folivorous and only two genera, *Varecia* and *Eulemur*, are considered mainly frugivorous^32–34^. This contrasting representation of frugivores in the New World and Madagascar is mirrored in other groups of mammals and birds^2,35^, and is considered a long-standing enigma in ecology^36,37^. There are 117 and 24 genera of fruit-eating birds and bats, respectively, in the New World, whereas there are merely five and three, respectively, in Madagascar^1^. Three non-mutually exclusive hypotheses have been put forward to explain the observed asymmetry between communities in dietary guild representation.

According to the food availability hypothesis, there should be a significant difference in the costs of frugivory due to temporal patterns of resource availability on different continents. In the New World, the high degree of overlap between periods of young leaf scarcity and low fruit availability would make it difficult for a frugivore to shift to a leafy diet during fruit shortages, thus making year-round frugivory obligatory. Here, during times of food scarcity, irregularly fruiting figs (*Ficus* spp.) act as keystone species that could allow frugivorous species to survive the lean period^5,38,39^. Conversely, extended lean periods, low predictability of fruiting, and the high frequency of cyclones have been hypothesized as driving forces that makes year-round frugivory challenging in Madagascar^40^. It has also been suggested that the contrasting abundance of certain keystone plants, such as figs, between continents contributes to the observed frugivore asymmetry^5,35,36^. The food availability hypothesis has been challenged by contradictory evidence of comparative studies and long-term phenological datasets in the New World^41,42^, while it stands as one of the major frameworks explaining lemur community structure in Madagascar^40,43^.

The historical hypothesis proposes that the lack of folivorous primates in the New World results from the inability of Platyrrhini (i.e., New World primates) ancestors to exploit the folivorous niche already occupied by other mammalian taxa, such as sloths^44,45^. On the contrary, that lemurs occupy a tremendous diversity of niches would have been possible due to the poor representation of other mammalian competitors in Madagascar. This idea, based on the rationale that primates arrived late in the New World and early in Madagascar relative to other mammals^44–46^, would be supported by evidence of niche compression in the most diverse New World primate communities^45^.

According to the nutritional hypothesis, if fruits contain enough nitrogen to meet primate protein requirements during gestation, lactation and weaning, there should be minimal selection to evolve a non-fruit diet and associated metabolic adaptations^47^. This would be particularly relevant for extant Neotropical and Malagasy primates that tend to be smaller in size relative to other primate radiations and thus should rely more heavily on food quality during key reproductive stages, i.e. income breeders^48–52^. This hypothesis is supported by a recent work that described higher fruit nitrogen content in the New World compared to Madagascar^47^. However, this study i) did not include samples from other continental areas occupied by primates; ii) relied on limited sample size and locations; and iii) did not control for factors such as spatial autocorrelation and sampling effort.

Here, we tested the robustness of the nutritional hypothesis by adding African and Asian sites to the comparison and by correcting previous shortcomings through the use of models accounting for different sampling efforts and non-independence of data. For this, we used a data-set of published and unpublished data on nitrogen content in fruits consumed by primates, as collated from 79 studies at 62 sites distributed across the New World, the Old World (Africa and Asia), and Madagascar. We first tested the expectation that primate communities in Africa and Asia are intermediate in terms of frugivorous guild representation between the New World (highest proportion) and Madagascar (lowest proportion). We used the proportion of frugivorous primate species and the average frugivory at a given site as measures of frugivory. In line with the nutritional hypothesis, we then tested the prediction that fruit nitrogen content will show the highest values at New World sites and the lowest values in Madagascar, while Old World sites will be intermediary. Since the nutritional hypothesis only makes sense if the consumers within different primate radiations have similar protein requirements, we also tested whether or not the overall diet (including all plant items) of species included in the analysis (representing all primate radiations) contained similar concentrations of proteins. Finally, we modelled fruit nitrogen to test whether it can explain geographical patterns of primate frugivory globally and within regions.

## Results

### Geographical pattern of primate frugivory

**Figure 1:**
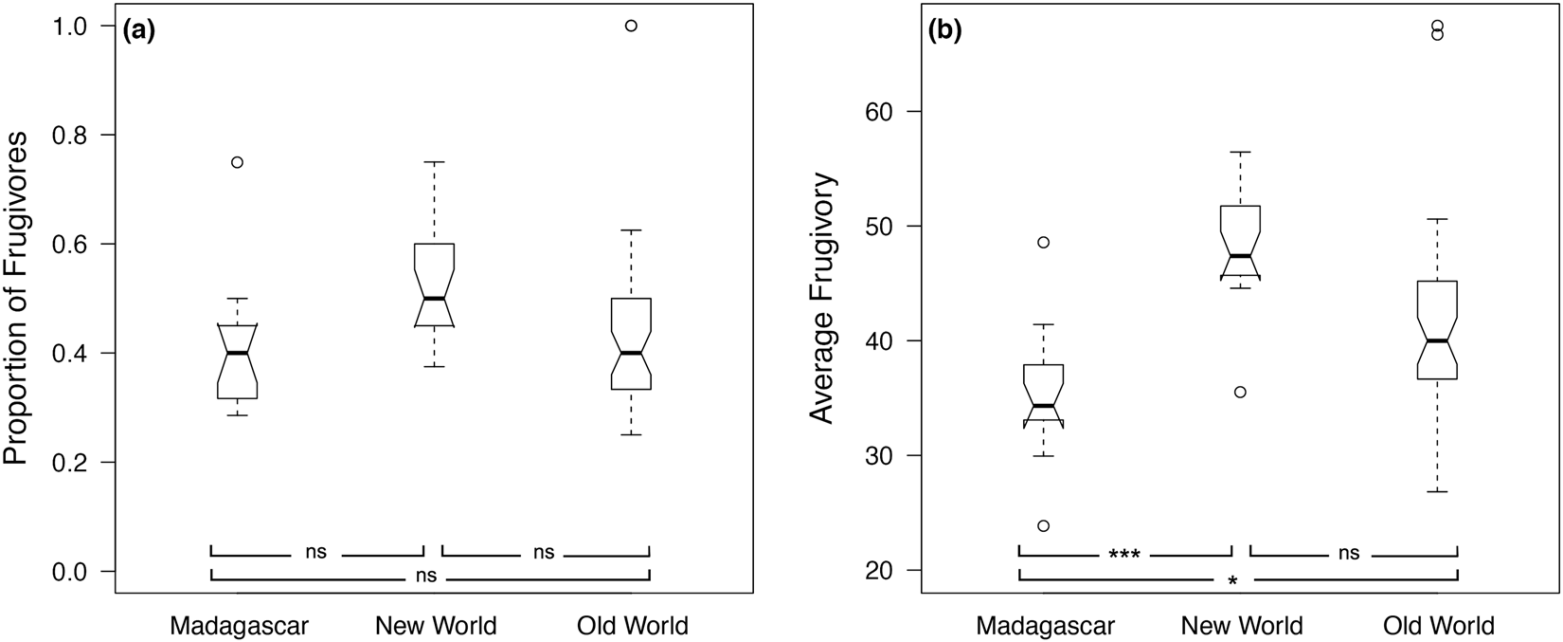
Notched boxplot representing the comparison in the proportion of frugivorous primates (**a**) and the average frugivory in primate diets (**b**) at 62 sites in Madagascar, New World, and Old World (raw data; without correction for autocorrelations). While the boxes encompass the interquartile range, the notches represent the 95% confidence interval of the median (central line). Proportion of frugivorous primates is represented as proportion of primate species with more than 50% fruits in their diet. *p < 0.05; ***p < 0.001; ns = non significant.

As expected, the degree of primate frugivory was higher in the New World and the Old World, and lower in Madagascar. The proportion of frugivorous species per site (i.e., proportion of species with more than 50% fruits in their diet) is lower in Madagascar but not significantly different from the New World (GLS_sp_ estimate ± SE: 0.07 ± 0.07, p = 0.37; ΔAIC of the null model = 3.15) or the Old World (0.05 ± 0.07, p = 0.48; Fig. 1). The average degree of frugivory (i.e., average proportion of fruits in diet), on the other hand, is significantly higher in the New World (12.04 ± 3.23, p < 0.001; ΔAIC of the null model = 9.69) and in the Old World (6.71 ± 2.97, p = 0.026) compared to Madagascar.

### Geographical pattern of fruit nitrogen and average nitrogen in primate diets

**Figure 2:**
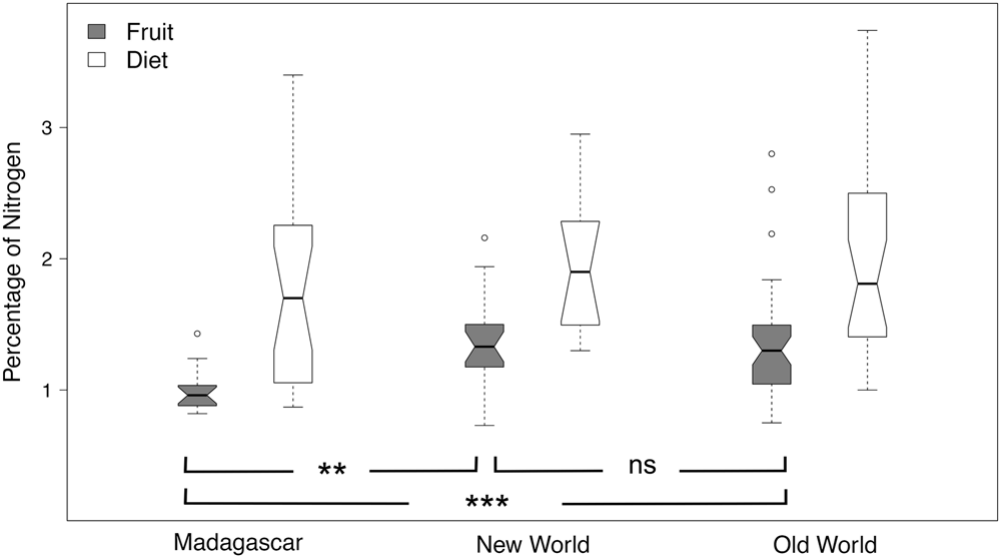
Notched boxplot representing the comparison in the nitrogen concentration in fruits and primate diet in Madagascar, New World, and Old World (raw data). While the boxes encompass the interquartile range, the notches represent the 95% confidence interval of the median (central line). Nitrogen is represented as percentage of dry matter. **p < 0.01; ***p < 0.001; ns = non significant.

Similar to the pattern of frugivory, fruit nitrogen content was higher in the New World and Old World, and lower in Madagascar. Our model estimated fruit nitrogen content to be higher at sites in the New World (GLS_sp_ estimate ± SE = 1.28% ± 0.10%), followed by the Old World (1.25% ± 0.08%) and Madagascar (0.98% ± 0.06%) (ΔAIC of the null model = 9.68; Fig. 2, Supplementary Table S1). Fruit nitrogen content in both the New World and the Old World was significantly higher than in Madagascar (Tukey post-hoc test: New World – Madagascar = 0.30 ± 0.10, p = 0.006; Old World – Madagascar = 0.28 ± 0.08, p = 0.001), whereas there was no significant difference between the two continental areas (New World – Old World = −0.03 ± 0.09, p = 0.95). These results were consistent with those obtained using the GLS not controlled for spatial autocorrelation (GLS_nsp_) (Supplementary Tables S2 and S3).

Conversely, nitrogen content in the overall diet of the primate species included in our analysis did not differ between New World, Old World, and Madagascar (Fig. 2, Supplementary Table S4). The Generalized Least Square model had a lower AIC than the corresponding null models that assumed no difference between the three areas (ΔAIC = 0.34). However, the model was not supported due to the higher number of parameters while standing within 2 units of AIC from the null model. Nitrogen content was estimated to be highest in the diet of New World primates (GLS estimate ± SE = 2.17% ± 0.22%), followed by lemurs (1.78% ± 0.12%) and Old World primates (1.75% ± 0.18%). These estimates were not significantly different between the three groups (Tukey post-hoc test: Old World - New World primates = −0.42 ± 0.24, p = 0.18; Old World primates - lemurs = −0.03 ± 0.18, p = 0.98; New World primates - lemurs = 0.39 ± 0.22, p = 0.20).

Despite the above differences, globally the proportion of frugivorous primate species and the average frugivory were uncorrelated with the protein concentrations in fruits at a given site (Supplementary Table S5). In both cases, the best model includes an interaction term between the three regions and the nitrogen content in fruits. However, considering the relationship separately for the three regions, fruit nitrogen is significantly positively related to the proportion of frugivores (GLS_sp_ estimate ± SE: 0.06 × 10^−1^ ± 0.05 × 10^−1^, p < 0.001) and average frugivory (0.23 ± 0.02, p < 0.001) in Madagascar, but shows no relationship in the New World (−0.06 × 10^−1^ ± 0.01 for the proportion of frugivores and −0.27 ± 0.49 for average frugivory), or in the Old World (−0.06 × 10^−1^ ± 0.05 × 10^−2^ for the proportion of frugivores and −0.23 ± 0.02 for average frugivory).

## Discussion

Our study supports the prediction of the nutritional hypothesis in explaining the lower proportion of frugivorous species in Madagascar primate communities compared with those on other continents^47^. The average frugivory of lemur communities was lower compared to New World and Asian-African (combined as Old World) primate communities. This contrasting representation of average frugivory is mirrored by the data on fruit proteins, since fruits at Malagasy sites showed lower average nitrogen content than those at Old World and New World sites. The variation between continents follows a similar pattern when the proportion of primate species that include more than 50% of fruits in their diet is used to estimate frugivory, although these differences do not reach statistical significance. However, contrary to the expectation, the degree of frugivory of the New World primate communities did not differ from Asian/African sites despite the highest representation of frugivorous primates revealed by other studies in the Neotropics^29,30^. This contrasts with recent analyses on birds indicating that present-day climate and forest productivity correlate with high proportion of frugivorous species in the Neotropics^37^. In birds, the high diversity of fleshy fruit plant species in both lowland and mountain regions in the Neotropics has been used to explain the high proportion of frugivorous species in habitats of both equatorial latitude regions of South America^53^. Since primate seed dispersal often co-occurs in plant families exhibiting bird dispersal^2^, we would expect that similar ecological factors may have also played a role for primates. The observation that this was not the case for our study sites suggests that different ecological constraints operate on primates and contributed to the observed pattern^5,36,44^. Sampling biases may have also been responsible for the lack of substantial differences in degree of frugivory between primates in the New World and Old World, both in terms of community composition and fruit protein content. For example, some of our New World study sites contain primate communities that are peripheral to the Amazon basin and only include a few species.

The overall lack of correlation between the degree of primate frugivory and fruit nitrogen concentrations indicates that evolutionary processes and contemporary ecological patterns are governed by multiple forces. The local representation of frugivorous species is likely to be profoundly influenced by factors such as population densities, species body mass, social systems and the number of species in a community. These factors might impose different constraints on the proportion of frugivorous primates than the radiation of species numbers over evolutionary time scales. However, the strong positive correlation between fruit protein and degree of frugivory for Madagascar but not for the other continents points to low fruit protein concentrations as a specific constraint on the island that has not become effective in other parts of the world. This echoes recent findings which indicate that folivorous primates select for high protein leaves only in forests where the average protein content in leaves is low^26^. Similarly, populations of arboreal marsupials do not appear to persist in areas where nutrient/toxin ratios fall below a threshold, while areas above that threshold appear constrained by a range of other factors that seem to play a role in the regulation of populations^54^.

According to the nutritional hypothesis, the average percentage of fruit nitrogen content in Madagascar (0.96%) is below the minimum nitrogen requirements threshold for primates (1.1%)^55^. This may have increased the selective pressures to evolve non-frugivorous diets and associated adaptations in lemurs^47^. The nutritional hypothesis is further corroborated by our control analysis that revealed similar nitrogen content in the overall diet of platyrrhines, catarrhines, and lemuriformes, which indicated compensation from other food categories when fruit nitrogen is scarce.

The main consequence of having to rely on food with relatively low nitrogen is that nutritional intake is limited by the ability to process enough food material, rather than by the scarcity of the nutrient itself^56,57^. In Madagascar, fruit consumption may also be limited by the availability of fruit trees that seem to be much smaller compared to those in other parts of the world, combined with more erratic fruiting patterns^40,43,58^. Under these conditions, animals may either shift to more proteinaceous food, such as leaves and/or invertebrates, or increase the amount of food processed. In fact, over-ingestion to meet protein requirements has been observed in frugivorous birds^59^ and some primates^10^ but see^60^, and a loss of body mass may even occur when protein intake is low despite an energy-rich diet^6^. In line with the nutritional hypothesis, the most frugivorous lemur genera, *Varecia* and *Eulemur* (family Lemuridae), are characterized by an “intake” strategy with some of the fastest food passage rates relative to body size recorded in primates^61,62^. Also, most lemurids are cathemeral, i.e. active over the 24 hours, an adaptation that may have helped to further increase both the amount of food processed and the nitrogen intake in animals lacking a specialized digestive system^63^.

It is important to note that the lack of global correlation with available nitrogen and other existing ecological processes may be a consequence of historical factors that could have amplified or hidden those effects. These include the evolutionary history of the fruiting trees, the presence or absence of other vertebrate competitors, the paleo-climate, the presence of dispersal barriers, or even human-induced extinctions^1,64,65^. For example, the high proportion of fruit-eating primates in the New World might be the result of the post-Pleistocene megafauna extinctions caused by the arrival of humans in Central and South America^66^. This idea appears to be supported by the reported extirpation of a large woolly monkey, *Brachyteles* sp., and the recent discovery of a subfossil giant platyrrhine^67,68^. This might explain the upper truncation in the body mass-diet relationship in New World primates. The hypothesis, however, is bound by the meager fossil record of extinct folivorous primates in the Americas as compared to other continents^69^. Historical events may have also played a major role in determining the current low diversity of frugivorous lemurs in Madagascar. A recent analysis shows that stem lineages leading to the *Indri* and *Avahi* clades (currently both fully folivorous) probably had a frugivorous diet, suggesting that frugivory was previously present in a more diverse array of lemur taxa compared to today^70^.

As a note of caution, however, we acknowledge that this idea remains exploratory until more data are collected on actual nutrient availability and intake among primates^71^. Despite the global scale of this analysis and the increased number of field sites over the last decade where primates are studied, sampling efforts on primate food are both taxonomically and geographically biased with efforts concentrated at relatively few sites and specific regions^72^. Another problem is in the difficulty of sampling the full breadth of the primate diet even for those species that have been longitudinally studied. This is particularly true for areas where fruiting plant patterns show multi-annual cycles and/or where plant diversity is relatively high^4,58,73^.

In summary, our results provide support to the hypothesis that lower availability of fruit proteins in Madagascar may not have provided an adequate protein intake to allow the evolution of frugivory among the radiations of the island’s endemic primates. Low protein availability in fruits may have also caused a more compelling relationship between the degree of frugivory in current lemur communities and fruit proteins available in their habitats, while other primate radiations appear less constrained by fruit proteins. As food characteristics influence the evolution of traits beyond dietary adaptations^74^, the deviating nutritional characteristics revealed by our analysis might complement the factors already identified^40,43^ as having broad implications for the evolution of life history traits of Madagascar’s biota.

## Materials and Methods

### Data-set

**Figure 3:**
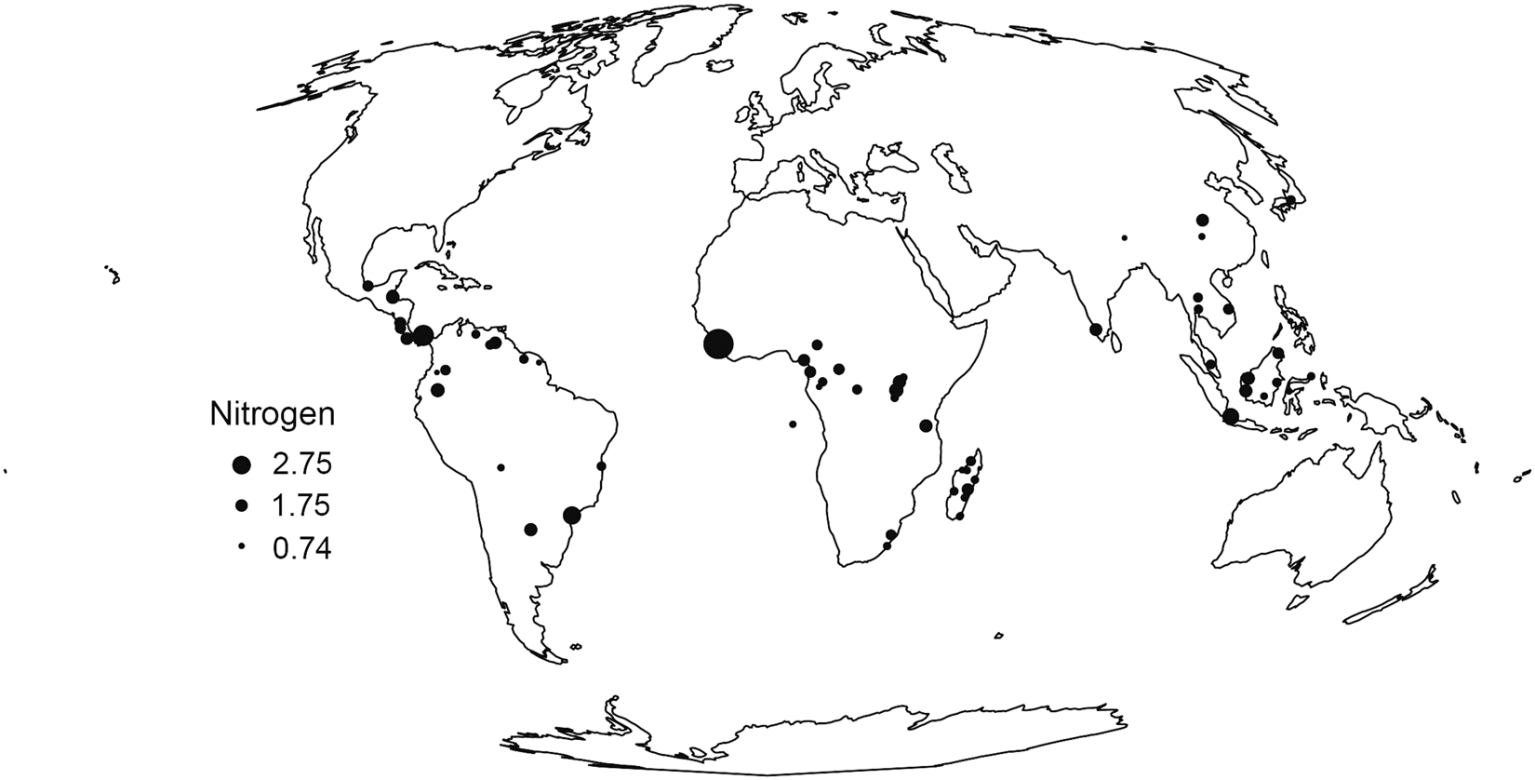
Map representing the 62 locations for which nitrogen records were available. Point size is proportional to the average nitrogen concentration in fruits per site. Studies are listed in Supplementary Table S1. The figure was created using “maptools” package in R v 3.3.2 (https://cran.r-project.org/).

We used published and unpublished data (Supplementary Table S1) to compile mean fruit nitrogen concentrations from 79 studies at 62 forest sites across three continental areas: Madagascar (15 studies), Old World (44 studies), and New World (20 studies) (Fig. 3). Primates comprise several large radiations: the Tarsiiformes (tarsiers) branched off at ~81 Mya, the split between the Catarrhini (Old World monkeys and apes) and the Platyrrhini (New World monkeys) dates back to ~43 Mya, and that between the Lemuriformes (Malagasy lemurs) and Lorisiformes (lorises, galagos, and pottos) has been estimated at ~69 Mya^75^. Tarsiers and some lorises are almost completely faunivorous. Fruit-eating amongst the remaining Lorisiformes is rare, with the exception of a handful of taxa^76^. As such, these two radiations of small-bodied primates are excluded from the analysis. We used a broad-scale of biogeographic regions to compare primate radiations at the taxonomic infraorder level. Thus, we combined Asian and African sites as Old World sites to represent the Catarrhini, while sites in Madagascar and in the New World represent the Lemuriformes and Platyrrhini radiations, respectively.

Given that no primates to our knowledge live in the dry savannas or spiny forests of the Americas, studies conducted in non-forest habitats or sites with more than nine consecutive dry months (defined as months with <100 mm rainfall) in the Old World and Madagascar were excluded from the analysis. This helps to keep the abiotic conditions more comparable amongst the three regions. We used nitrogen concentrations rather than crude protein in our comparison because different conversion factors from nitrogen to crude protein have been suggested^77^. Only studies reporting total nitrogen measured with the Kjeldahl method were used in the analysis^78^.

We considered the nitrogen concentration in fruits consumed by primates as representative samples of the nitrogen concentrations for all fruits available at each site. Our assumption is based on the observation that in most studies addressing protein selection in fruits, there were no reported significant differences between fruits eaten and not eaten by primates^47,79,80^. Thus, to compile fruit data at a given site we used both general sampling of all fruits (both ripe and unripe) and/or fruits eaten by primates.

### Analyses

#### Geographical pattern of primate frugivory

To test the expectation that primate frugivory is higher in the Neotropics than in the Old World and Madagascar, we first compared frugivory between the three regions expressed both as the proportion of frugivores (>50% of fruit in the diet) and as average frugivory (average proportion of fruits in the diet) at the 62 sites present in our data-set. The diets of all primate species at the 62 sites were determined from a comprehensive literature survey using the All The World Primates’ database^81^. We followed previous authors who define *frugivore* as an animal whose diet is composed of more than 50% fruits^1^. We used the average when more than one study was available at a given site for a species. When no information was available about a species at a given site, we used species information from a different study site. Because study sites are unevenly distributed and the similarity in frugivory between two sites can reflect their geographical distance, we controlled for spatial autocorrelation in the models. We first ran a null model (only-intercept models) with the proportion of frugivores and average frugivory as dependent variables, and tested the spatial autocorrelation in the residuals using Moran’s autocorrelation coefficient (often denoted as Moran I). Moran I is an extension of Pearson product-moment correlation coefficient to a univariate series^82^. Since Moran I was significant in both cases (proportion of frugivores: Moran I = 0.07; Expected value = −0.01; p = 0.046; average frugivory: Moran I = 0.34; Expected value = −0.01; p < 0.001), we ran Generalized Least Square Models (GLS) controlled for spatial autocorrelation (GLS_sp_)^83^. We tested the gaussian (corGaus), the exponential (corExp) and the spheric (corSpher) correlation structure, and selected the former as the one giving the lowest Akaike Information Criterion (AIC proportion of frugivores: Gauss = −164.08; Exp = −136.25; Spher = −136.57; AIC average frugivory: Gauss = 442.22; Exp = 461.37; Spher = 461.76). To assess whether the model complexity was justified by an increase in goodness of fit, we compared the two GLS_sp_ with their corresponding null models using AIC. Models were considered supported when ΔAIC < 2 (ΔAIC = difference in AIC from the best model). Following Pinheiro & Bates^84^ all GLSs that were compared through AIC were fitted using Maximum Likelihood (ML), whereas the final GLSs were fitted using the Restricted Maximum Likelihood (REML).

#### Geographical pattern of fruit nitrogen and average nitrogen in primate diets

We compared the mean nitrogen content in fruits between the three regions using individual studies as our unit of analysis. Since large variations in the nutritional content of fruits have been reported by different studies even within the same site^85^, we considered the mean of each study as a separate datum. However, because this choice may lead to pseudo-replication, as well as spatial autocorrelation, we controlled for their spatial non-independence. As above, we tested the spatial autocorrelation in the residuals using Moran’s autocorrelation coefficient. Since Moran I was almost significant (Moran I = 0.06; Expected value = −0.01; p = 0.057), we ran two Generalized Least Square Models (GLS), one non-controlled for spatial autocorrelation (GLS_nsp_) and one controlled for spatial autocorrelation (GLS_sp_)^83^. For the latter, we tested the gaussian (corGaus), the exponential (corExp) and the spheric (corSpher) correlation structure, and selected the latter as the one giving the lowest Akaike Information Criterion (AIC: Gauss = 61.06; Exp = 60.69; Spher = 59.02). In order to control for the unequal sampling effort at different locations (sample size over which nitrogen was averaged), we weighted the two GLS models by the sample size per site. Because weights expressed as sample size may over-emphasize the importance of certain records, weights were transformed using a parameter Omega^86^. Omega is an elevation factor that ranges between 1 (the original sample sizes are used as weights) and 1/100 (all records are weighted equally). To estimate the Omega parameter, the GLS were repeatedly fitted using increasing values of Omegas, and used AIC to identify the best fitting model. The Omega corresponding to the lowest AIC was equal to 0.64 in GLS_sp_ and 0.67 in GLS_nsp_. To assess whether model complexity was justified by an increase in goodness-of-fit, we compared the final GLS_nsp_ and GLS_sp_ with corresponding null models using AIC.

To test whether consumers of different primate radiations have similar protein requirements, we compared the overall dietary nitrogen between the three regions using primate species as the unit of analysis. Because the residuals of statistical models using species as units of analysis can be phylogenetically autocorrelated, we first fitted an ANOVA model and tested its residuals for phylogenetic autocorrelation using Pagel’s lambda^87^. We used the consensus phylogenetic tree from the 10kTrees phylogenies project based on the taxonomy of Wilson and Reeder^88^ (version 3; http://10ktrees.nunnlab.org/Primates/downloadTrees.php). Pagel’s lambda was 6.63 × 10^−5^ and not significantly different from zero (i.e. no autocorrelation), therefore there was no need to correct the model by accounting for phylogenetic autocorrelation. Similar to the previous model, we ran the ANOVA as a GLS weighted by the sample size and selected the Omega by using AIC and ML, and estimated the coefficients using the REML. For this model, Omega was equal to zero (i.e. all studies were weighted equally). The final GLS was compared with a corresponding null model using AIC.

In the models described above, we tested all comparisons between the three regions using the Tukey post-hoc test. All analyses were performed in R 3.3.0^89^ using “nlme” package^90^ for running the GLS models, “spdep”^91^ for testing the Moran I, “phytools”^92^ for testing Pagel’s lambda, “ape”^93^ for loading the phylogenetic tree, and “multcomp”^94^ for the Tukey post-hoc comparisons of the GLS models.

## Electronic supplementary material

Tables S1-S5
