# Supplementary material for: Low Levels of Fruit Nitrogen as Drivers for the Evolution of Madagascar’s Primate Communities
Source: Sci Rep. 2017 Oct 31;7:14406. doi: 10.1038/s41598-017-13906-y (PMC5663947; doi:10.1038/s41598-017-13906-y)
Supplement: Supplementary file 1 — Tables S1-S5 [file 41598_2017_13906_MOESM1_ESM.pdf]

Low Levels of Fruit Nitrogen as Drivers for the Evolution of Madagascar's Primate Communities

Giuseppe Donati, Luca Santini, Timothy M. Eppley, Summer J. Arrigo-Nelson, Michela Balestri, Sue Boinski, An Bollen, LeAndra L. Bridgeman, Marco Campera, Valentina Carrai, Mukesh K. Chalise, Abigail Derby Lewis, Gottfried Hohmann, Margaret F. Kinnaird, Andreas Koenig, Martin M. Kowalewski, Petra Lahann, Matthew R. McLennan, Anna K.I. Nekaris, Vincent Nijman, Ivan Norscia, Julia Ostner, Sandra Y. Polowinsky, Oliver Schülke, Christoph Schwitzer, Pablo R. Stevenson, Mauricio G. Talebi, Chia L. Tan, Irene Tomaschewski, Erin R. Vogel, Patricia C. Wright, Jörg U. Ganzhorn

Supplementary Table S1. Nitrogen concentrations of fruits at various sites in the Old World, Madagascar, and the New World. Nitrogen concentrations in fruits have been calculated either by general sampling (GS) or using samples consumed by a specific primate species (PF). Taxonomic source as for Mittermeier et al. 2013 [84].

**Site**	**Country**	**Mean % Nitrogen concentration in fruits (sample size)**	**Sampling**	**Source**
**Old World**				
Campo	Cameroon	1.16 (13)	PF: *Gorilla gorilla gorilla*, *Cercocebus torquatus*	1, 2
Lope	Gabon	0.84 (46)	PF: *Gorilla gorilla gorilla*	3
Bai Hokou	Central African Republic	1.36 (22)	PF: *Gorilla gorilla gorilla*	4
Bwindi	Uganda	1.53 (32)	PF: *Gorilla beringei beringei*	5, 6, 7
Ipassa	Gabon	1.15 (8)	PF: *Pan troglodytes troglodytes*	8
Oribi Gorge	South Africa	1.05 (26)	GS	9
Kibale	Uganda	1.50 (59)	PF: *Pan troglodytes schweinfurthii* + GS	10, 11
Gashaka	Nigeria	1.30 (73)	PF: *Pan troglodytes ellioti*	11
Tai	Ivory Coast	0.94 (44)	PF: *Pan troglodytes verus*	11
Salonga	Democratic Republic of Congo	1.23 (73)	PF: *Pan paniscus*	11
Tiwai	Sierra Leone	1.74 (29)	PF: *Piliocolobus badius*, *Procolobus verus*, *Colobus polykomos* + GS	12, 13
KwaZulu-Natal	South-Africa	1.31 (30)	GS	14
Udzungwa Mountains	Tanzania	1.52 (44)	PF: *Cercocebus sanjei*	15
Korup	Cameroon	1.44 (3)	PF: *Piliocolobus preussi*	16
Nyungwe	Rwanda	1.07 (23)	GS	17
Bulindi	Uganda	1.03 (41)	PF: *Pan troglodytes schweinfurthii*	18

Muara Gembong	Indonesia	1.84 (8)	PF: *Trachypithecus auratus*	19
Kuala Lompat	Malaysia	1.21 (8)	GS	20
Sepilok	Malaysia	1.34 (18)	PF: *Presbytis rubicunda chrysea* + GS	20, 21
Kutai	Indonesia	0.90 (39)	PF: *Pongo pygmaeus morio, Presbytis hosei canicrus*	21, 22
Sibaliw	Philippines	0.81 (49)	GS	Luft dataset S1
Gunung Palung	Indonesia	1.52 (85)	PF: *Pongo pygmaeus wurmbii*	23
Tuanan	Indonesia	0.99 (180)	PF: *Pongo pygmaeus wurmbii*	24
Tanjung Puting	Indonesia	0.83 (22)	PF: *Nasalis larvatus larvatus*	25
Fanjing Mountain	China	0.91 (5)	PF: *Rhinopithecus brelichi*	25
Tangkoko	Indonesia	1.05 (58)	PF: *Macaca nigra*	Kinnaird dataset S1
Phu Khieo	Thailand	1.22 (46)	PF: *Macaca assamensis assamensis*	Schülke dataset S1
Khao Yai	Thailand	1.16 (29)	PF: *Hylobates lar entelloides*	26
Kon Ka Kihn	Vietnam	1.33 (11)	PF: *Pygathrix cinerea*	27
Qianjiaping	China	1.48 (14)	PF: *Rhinopithecus roxellana hubeiensis*	28
Lore Lindu	Indonesia	0.86 (13)	PF: *Macaca tonkeana*	29
Silent Valley	India	1.49 (13)	PF: *Macaca silenus*	30
Sikkim	India	0.75 (19)	GS	31
Aichi	Japan	1.22 (5)	PF: *Macaca fuscata fuscata*	32
**Madagascar**				
Mandena	Madagascar	0.90 (207)	PF: *Cheirogaleus medius* + *Cheirogaleus major* + *Eulemur collaris* + *Hapalemur meridionalis* + *Microcebus ganzhornii* + GS	33, 34, 35, Campera & Balestri dataset S1
Ste Luce	Madagascar	0.90 (267)	PF: *Cheirogaleus medius* + *Eulemur collaris* + GS	33, 36, Campera & Balestri

				dataset S1
Kirindy	Madagascar	0.97 (53)	PF: *Propithecus verreauxi* + GS	33, 37, 38
Ranomafana	Madagascar	1.00 (51)	PF: *Propithecus edwardsi, Microcebus rufus*	33, 39
Sahamalaza	Madagascar	1.24 (67)	GS	33
Anjamena	Madagascar	0.88 (25)	PF: *Eulemur mongoz*	40
Tsinjoarivo	Madagascar	1.43 (22)	PF: *Propithecus diadema*	41
Ampijoroa	Madagascar	0.99 (3)	PF: *Eulemur fulvus*	42
Betampona	Madagascar	1.06 (84)	PF: *Varecia variegata variegata*	43
**New World**				
Los Tuxtlas	Mexico	1.34 (11)	PF: *Alouatta palliata mexicana*	44
Cockscomb Basin	Belize	1.33 (16)	PF: *Alouatta pigra*	45
Barro Colorado Island	Panama	1.66 (14)	GS	46, 47
Hato Masaguaral	Venezuela	1.12 (9)	PF: *Alouatta macconnelli*	48
Lago Guri	Venezuela	1.47 (19)	PF: *Pithecia pithecia* + 3 fruits not eaten	49
Nouragues	French Guiana	0.79 (14)	GS	50
San Cayetano / Isla Brasilera	Argentina	1.60 (3)	PF: *Alouatta caraya*	33, 51
Lemos Maia	Brasil	1.18 (22)	PF: *Callicebus moloch*	52
Finca la Luz	Nicaragua	1.41 (14)	PF: *Alouatta palliata palliata*	53
Monkey river	Belize	1.56 (9)	PF: *Alouatta pigra*	54
Guarayos	Bolivia	0.96 (28)	PF: *Ateles chamek*	55
Lomas Barbudal	Costa Rica	1.33 (64)	PF: *Cebus capucinus capucinus*	33
Tinigua	Columbia	1.28 (53)	PF: *Lagothrix lagotricha*	33
Raleighvallen	Suriname	1.17 (13)	PF: *Sapajus apella apella*	33
Yasuni'	Ecuador	1.59 (33)	PF: *Alouatta seniculus juara* + NPF	33
Parque Estadual Carlos Botelho	Brasil	1.94 (4)	PF: *Brachyteles arachnoides*	56
Finca Merenberg	Columbia	0.73 (27)	GS	57
El Caura	Venezuela	1.19 (44)	PF: *Ateles belzebuth*	58

Corcovado	Costa Rica	1.48 (10)	GS	59

Supplementary Table S2. Model results for the Generalized Least Square model comparing the nitrogen in fruits between Madagascar, Old World and New World, not controlling for spatial autocorrelation (GLS_{nsp}), and corresponding null model (only-intercept model). SE = standard error; ΔAIC = difference in AIC from the best model.

**Model**	**Terms**	**Estimate**	**SE**	**p-value**	**ΔAIC**
GLS_{nsp}	Intercept (Madagascar)	0.976	0.063	0.000	0
	Old World	0.276	0.079	0.003	
	New World	0.304	0.098	0.001	
Null model	Intercept	1.179	0.036	0.000	8.12

Supplementary Table S3. Tukey post-hoc test for the Generalized Least Square model (GLS_{nsp}) results for the Generalized Least Square model comparing the nitrogen in fruits between Madagascar, Old World and New World, not controlling for spatial autocorrelation.

**Post-hoc comparison**	**Estimate**	**SE**	**p-value**
New World - Madagascar	0.304	0.098	0.005
Old World - Madagascar	0.276	0.079	0.001
Old World – New World	-0.028	0.088	0.945

Supplementary Table S4. Mean nitrogen concentration of all plant food items consumed by the primate species in the Old World, Madagascar, and the New World.

**Species**	**Site**	**Mean % Nitrogen concentration in food (sample size)**	**Source**
**Old World**			
Cercocebus sanjei	Udzungwa Mountain	2.07 (96)	15
Cercocebus torquatus	Campo	1.40 (5)	2
Cercopithecus ascanius schmidti	Kibale	2.70 (18)	10
Cercopithecus mitis ssp.	Kibale & Zomba Plateau	1.85 (82)	10, 60
Piliocolobus preussi	Korup	1.43 (17)	16
Colobus guereza	Kakamega	3.30 (22)	61
Colobus polykomos	Tiwai	2.90 (25)	12

Gorilla beringei beringei	Bwindi & Virunga	1.94 (126)	6, 7
Gorilla gorilla gorilla	Lope, Bai Hokou & Campo	1.63 (169)	1, 3, 4
Hylobates lar entelloides	Khao Yai	1.00 (16)	26
Lophocebus albigena	Kibale	2.60 (18)	10
Macaca assamensis assamensis	Phu Khieo	1.22 (58)	Schülke dataset S1
Macaca fuscata fuscata	Aichi	1.27 (16)	32
Macaca nigra	Tangkoko	1.05 (58)	Kinnaird dataset S1
Nasalis larvatus larvatus	Labuk Bay, Tanjung Puting & Lower Kinabatangan	1.41 (39)	62, 63, 64
Pan troglodytes schweinfurthii	Kibale, Bulindi	1.43 (45)	2, 18
Pongo pygmaeus ssp.	Kutai, Tanjung, Cabang Panti, & Tuanan	1.26 (385)	22, 23, 24, 65
Presbytis siamensis siamensis	Kuala Lompat	1.40 (12)	20
Presbytis rubicunda chrysea	Sepilok & Lower Kinabatangan	2.04 (36)	21, 66
Piliocolobus tephrosceles	Kibale	3.63 (63)	67
Procolobus verus	Tiwai	2.80 (5)	68, 69
Pygathrix cinerea	Kon Ka Kihn	1.81 (33)	27
Rhinopithecus bieti	Mt Longma	1.76 (9)	70
Rhinopithecus roxellana hubeiensis	Qianjiaping	1.80 (117)	28
Semnopithecus hector	Rajaji	2.40 (16)	71
Trachypithecus auratus	Java	2.00 (13)	72
**Madagascar**			
Avahi peyrierasi	Ranomafana	2.50 (5)	73
Avahi meridionalis	Ste Luce	1.14 (47)	74
Cheirogaleus major	Mandena	0.90 (77)	34
Cheirogaleus medius	Mandena & Ste Luce	0.87 (108)	34, 36
Eulemur collaris	Mandena & Ste Luce	1.01 (176)	36, 75, Campera & Balestri dataset S1
Eulemur flavifrons	Sahamalaza	1.60 (88)	33
Eulemur fulvus	Ampijoroa	2.07 (8)	42
Eulemur macaco	Ampasikely	1.70 (23)	76
Eulemur mongoz	Anjamena	1.10 (46)	40
Eulemur rufifrons	Kirindy	1.00 (20)	37

Hapalemur alaotrensis	Alaotra	2.10 (15)	77
Hapalemur aureus	Ranomafana	3.20 (63)	33
Hapalemur griseus	Ranomafana	3.40 (40)	33
Hapalemur meridionalis	Mandena	1.37 (141)	35, 78
Prolemur simus	Ranomafana	2.30 (141)	33
Indri indri	Mantadia	1.70 (10)	79
Lepilemur ruficaudatus	Kirindy	2.40 (194)	80
Microcebus ganzhornii	Mandena	0.90 (77)	33, 34
Microcebus rufus	Ranomafana	0.90 (12)	39
Propithecus diadema	Tsinjoarivo & Mantadia	2.30 (87)	41, 79
Propithecus edwardsi	Ranomafana	2.00 (392)	33
Propithecus verreauxi	Kirindy	2.21 (260)	33, 38
Varecia variegata variegata	Betampona	1.30 (122)	43
**New World**			
Alouatta caraya	San Cayetano & Isla Brasilera	2.59 (46)	33, 51
Alouatta palliata mexicana	Finca la Luz, Los Tuxlas, Barro Colorado Island & La Suerte	2.25 (134)	44, 46, 81
Alouatta pigra	Pantanos de Centla, Monkey river, Cockscomb Basin	2.95 (173)	45, 54, 82
Alouatta seniculus juara	Yasuni'	2.30 (124)	33
Alouatta macconnelli	Hato Masaguaral	2.35 (37)	48
Ateles belzebuth	El Caura	1.35 (49)	58
Ateles chamek	Guarayos Forest Reserve	1.64 (44)	55
Ateles geoffroyi ornatus	Barro Colorado Island	1.90 (4)	46
Brachyteles arachnoides	Parque Estadual Carlos Botelho	2.20 (10)	56
Callicebus moloch	Mata Atlantica	1.60 (32)	52
Cebus capucinus	Barro Colorado Island & Barbudal	1.39 (68)	33, 46
Lagothrix lagotricha	Tinigua	1.30 (53)	83

Supplementary Table S5. Selection of best fit models in the generalized least square regression testing the effect of regions and fruit nitrogen (N) on the proportion of frugivorous primates and the average frugivory.

Model	df	AIC	Delta
**pF50~N*Region**	**8**	**-176.984**	**0**
pF50~1	3	-164.079	12.904
pF50~N	4	-162.446	14.538
pF50~Region	5	-160.922	16.062
**aveFruit~N*Region**	**8**	**416.228**	**0**
aveFruit~Region	5	432.531	16.303
aveFruit~1	3	442.221	25.993
aveFruit~N	4	444.112	27.885

References

1. Calvert, J. J. Food selection by western gorillas (*G. g. gorilla*) in relation to food chemistry. *Oecologia* **65**, 236–246 (1985).
2. Mitani, M. *Cercocebus torquatus*: adaptive feeding and ranging behaviors related to seasonal fluctuations of food resources in the tropical rain forest of south-western Cameroon. *Primates* **30**, 307–323 (1989).
3. Rogers, M. E., Maisels, F., Williamson, E. A., Fernandez, M. & Tutin, C. E. G. Gorilla diet in the Lope Reserve, Gabon. *Oecologia* **84**, 326–339 (1990).
4. Remis, M. J., Dierenfeld, E. S., Mowry, C. B. & Carroll, R. W. Nutritional aspects of western lowland gorilla (*Gorilla gorilla gorilla*) diet during seasons of fruit scarcity at Bai Hokou, Central African Republic. *Int. J. Primatol.* **22**, 807–836 (2001).
5. Ganas, J. Foraging strategies of mountain gorillas (*Gorilla beringei beringei*) in Bwindi Impenetrable National Park, Uganda. PhD Thesis, Universität Leipzig (2007).
6. Rothman, J. M. *et al*. Nutritional chemistry of foods eaten by gorillas in Bwindi Impenetrable National Park, Uganda. *Am. J. Primatol.* **68**, 675–691 (2006).
7. Rothman, J. M., Plumptre, A. J., Dierenfeld, E. S. & Pell, A. N. Nutritional composition of the diet of the gorilla (*Gorilla beringei*): a comparison between two mountain habitats. *J. Trop. Ecol.* **23**, 673–682 (2007).
8. Hladik, C. M. Chimpanzees of Gabon and chimpanzees of Gombe: some comparative data on the diet. *Primate Ecology: Studies of Feeding and Ranging Behaviour in Lemurs, Monkeys and Apes* (ed. Clutton-Brock, T. H.) 481–501 (Academic Press, 1977).
9. Voigt, F. A. *et al*. A comparison of morphological and chemical fruit traits between two sites with different frugivore assemblages. *Oecologia* **141**, 94–104 (2004).

10. Conklin-Brittain, N. L., Wrangham, R. W. & Hunt, K. D. Dietary response of chimpanzees and cercopithecines to seasonal variation in fruit abundance. II. Macronutrients. *Int. J. Primatol.* **19**, 971–998 (1998).

11. Hohmann, G. *et al*. Plant food consumed by *Pan*: exploring the variation of nutritional ecology across Africa. *Am. J. Phys. Anthropol.* **141**, 476–485 (2010).

12. Dasilva, G. L. Diet of *Colobus polykomos* on Tiwai Island: selection of food in relation to its seasonal abundance and nutritional quality. *Int. J. Primatol.* **15**, 655–680 (1994).

13. Davies, A. G., Oates, J. F. & Dasilva, G. L. Patterns of frugivory in three West African colobine monkeys. *Int. J. Primatol.* **20**, 327–357 (1999).

14. Wilson, A. L. & Downs, C. T. Fruit nutritional composition and non-nutritive traits of indigenous South African tree species. *S. Afr. J. Bot.* **78**, 30–36 (2012).

15. McCabe, G. M., Fernández, D. & Ehardt, C. L**.** Ecology of reproduction in Sanje mangabeys (*Cercocebus sanjei*): dietary strategies and energetic condition during a high fruit period. *Am. J. Primatol.* 75, 1196–1208 (2013).

16. Usongo, L. I. & Amubode, F. O. Nutritional ecology of Preuss's red colobus monkey (*Colobus badius preussi* Rahm 1970) in Korup National Park, Cameroon. *Afr. J. Ecol.* **39**, 121–125 (2001).

17. Sun, C., Moermond, T. C. & Givnish, T. J. Nutritional determinants of diet in three turacos in a tropical montane forest. *Auk* **114**, 200–211 (1997).

18. McLennan, M. R. & Ganzhorn, J. U. Nutritional characteristics of wild and cultivated foods for chimpanzees (*Pan troglodytes*) in agricultural landscapes. *Int. J. Primatol.* 10.1007/s10764-016-9940-y (2017).

19. Supriatna, J. A. T. N. A. *et al*. Chemical analysis of food plant parts of two sympatric monkeys (*Presbytis aurata* and *Macaca fascicularis*) in the mangrove forests of Muara Gembong, west Java. *Symposium on Mangrove Management: Its Ecological and Economical Considerations* (eds. Soerianegara, I. *et al*.) 161-169 (SEAMEO-Biotrop: Biotrop Special Publication No. 37, 1988).

20. Davies, A. G., Bennet, E. L. & Waterman, P. G. Food selection by two south-east Asian colobine monkeys (*Presbytis rubicunda* and *Presbytis melalophos*) in relation to plant chemistry. *Biol. J. Linn. Soc.* **34**, 33–56 (1988).

21. Waterman, P. G. & Kool, K. M. Colobine food selection and plant chemistry. *Colobine Monkeys: Their Ecology, Behaviour and Evolution* (eds. Davies, A. G. & Oates, J. F.) 251–284 (Cambridge University Press, 1994).

22. Leighton, M. Modeling dietary selectivity by Bornean orangutans: evidence for integration of multiple criteria in fruit selection. *Int. J. Primatol.* **14**, 257–314 (1993).

23. Vogel, E. R. *et al*. Bornean orangutans on the brink of protein bankruptcy. *Biol. Lett.* **8**, 333–336 (2012).

24. Vogel, E. R. *et al*. Nutritional ecology of wild Bornean orangutans (*Pongo pygmaeus wurmbii*) in a peat swamp habitat: effects of age, sex, and season. *Am. J. Primatol*. 10.1002/ajp.22618 (2016).

25. Nijboer, J. *et al*. 1997 Chemical composition of Southeast Asian colobine foods. Proceeding of the second conference of the nutrition advisory group. AZA (1997).

26. Kanwatanakid-Savini, C., Poonswad, P. & Savini, T. An assessment of food overlap between gibbons and hornbills. *Raffles B. Zool.* **57**, 189–198 (2009).

27. Tinh, N. T., Long, H. T., Van Tuan, B., Vy, T. H. & Tam, N. A. The feeding behaviour and phytochemical food content of grey-shanked douc langurs (*Pygathrix cinerea*) at KonKaKinh National Park, Vietnam. *Vietnamese Journal of Primatology* **2**, 25–35 (2012).

28. Liu, X., Stanford, C. B., Yan, J., Yao, H. & Li, Y. Foods Eaten by the Sichuan snub-nosed monkey (*Rhinopithecus roxellana*) in Shennongjia National Nature Reserve, China, in relation to nutritional chemistry. *Am. J. Primatol.* **75**, 860–871 (2013).

29. Riley, E. P., Tolbert, B. & Farida, W. R. Nutritional content explains the attractiveness of cacao to crop raiding Tonkean macaques. *Curr. Zool.* **59**, 160–169 (2013).

30. Krishnadas, M. The response of the frugivorous lion-tailed macaque (*Macaca silenus*) to a period of fruit scarcity. *Am. J. Primatol.* **73**, 1250–1260 (2011).

31. Sundriyal, M. & Sundriyal, R. C. Wild edible plants of the Sikkim Himalaya: nutritive values of selected species. *Econ. Bot.* **58**, 286–299 (2004).

32. Jaman, M. F., Huffman, M. A. & Takemoto, H. The foraging behavior of Japanese macaques *Macaca fuscata* in a forested enclosure: effects of nutrient composition, energy and its seasonal variation on the consumption of natural plant foods. *Curr. Zool.* **56**, 198–208 (2010).

33. Ganzhorn, J. U. *et al*. Possible fruit protein effects on primate communities in Madagascar and the Neotropics. *PLoS One* **4**, e8253 (2009).

34. Lahann, P. Feeding ecology and seed dispersal of sympatric cheirogaleid lemurs (*Microcebus murinus*, *Cheirogaleus medius*, *Cheirogaleus major*) in the littoral rainforest of south-east Madagascar. *J. Zool.* **271**, 88–98 (2007).

35. Eppley, T. M., Donati, G. & Ganzhorn, J. U. Determinants of terrestrial feeding in an arboreal primate: the case of the southern bamboo lemur (*Hapalemur meridionalis*). *Am. J. Phys. Anthropol.* **161**, 328–342 (2016).

36. Bollen, A. Fruit characteristics: fruit selection, animal seed dispersal and conservation matters in the Sainte Luce forests. *Biodiversity, Ecology, and Conservation of Littoral Ecosystems in the Region of Tolagnaro (Fort Dauphin), Southeastern Madagascar* (eds. Ganzhorn, J. U., Goodman, S. M. & Vincelette, M.) 127–145 (Smithsonian Institution Press, 2007).

37. Bollen, A. *et al*. An intersite comparison on fruit characteristic. In *Tropical Fruits and Frugivores: The Search for Strong Interactors* (eds. Dew, J. L. & Boubli, J. P.) 93–119 (Springer, 2005).

38. Norscia, I., Carrai, V., Borgognini-Tarli, S. M. Influence of dry season and food quality and quantity on behavior and feeding strategy of *Propithecus verreauxi* in Kirindy, Madagascar. *Int. J. Primatol.* **27**, 1001–1022 (2006).

39. Atsalis, S. Diet of the brown mouse lemur (*Microcebus rufus*) in Ranomafana National Park. *Int. J. Primatol.* **20**, 193–229 (1999).

40. Curtis, D. J. Diet and nutrition in wild mongoose lemurs (*Eulemur mongoz*) and their implications for the evolution of female dominance and small group size in lemurs. *Am. J. Phys. Anthropol.* **124**, 234–247 (2004).

41. Irwin, M. T., Raharison, J. L., Raubenheimer, D., Chapman, C. A. & Rothman, J. M. Nutritional correlates of the "lean season": effects of seasonality and frugivory on the nutritional ecology of diademed sifakas. *Am. J. Phys. Anthropol.* **153**, 78–91 (2014).

42. Sato, H., Ichino, S. & Hanya, G. Dietary modification by common brown lemurs (*Eulemur fulvus*) during seasonal drought conditions in western Madagascar. *Primates* **55**, 219–230 (2014).

43. Schmidt, D. A. *et al*. Nutrient composition of plants consumed by black and white ruffed lemurs, *Varecia variegata*, in the Betampona Natural Reserve, Madagascar. *Zoo Biol.* **29**, 375–396 (2010).

44. Estrada, A. & Coates-Estrada, R. Frugivory in howling monkeys (*Alouatta palliata*) at Los Tuxtlas: dispersal and fate of seeds. *Frugivores and Seed Dispersal* (eds. Estrada, A. & Fleming, T. H.) 93–104 (Dr. W. Junk Publishers, 1986).

45. Silver, S. C., Ostro, L. E. T., Yeager, C. P. & Dierenfeld, E. S. Phytochemical and mineral components of food consumed by black howler monkeys (*Alouatta pigra*) at two sites in Belize. *Zoo Biol.* **19**, 95–109 (2000).

46. Hladik, C. M. *et al*. Le régime alimentaire des Primates de l'Ile de Barro-Colorado (Panama). Résultats des analyses quantitatives. *Folia Primatol.* **16**, 85–122 (1971).

47. Milton, K., Van Soest, P. J. & Robertson, J. B. Biology digestive efficiencies of wild howler monkeys. *Physiol. Zool.* **53**, 402–409 (1980).

48. Oftedal, O. T. The nutritional consequences of foraging in primates: the relationship of nutrient intake to nutrient requirements. *Phil. Trans. Roy. Soc. Lond. B.* **334**, 161–170 (1991).

49. Norconk, M. A. & Conklin-Brittain, N. L. Variation on frugivory: the diet of Venezuelan white-faced sakis. *Int. J. Primatol.* **25**, 1–26 (2004).

50. Simmen, B. & Sabatier, D. Diets of some French Guianan primates: food composition and food choices. *Int. J. Primatol.* **17**, 661–693 (1996).

51. Zunino, G. E. Hábitat, dieta y actividad del mono aullador negro (*Alouatta caraya*) en el noreste de la Argentina. *Boletín Primatológico Latinoamericano* **1**, 75–97 (1989).

52. Heiduck, S. Food choice in masked titi monkeys (*Callicebus personatus melanochir*): Selectivity or opportunism? *Int. J. Primatol.* **18**, 487–502 (1997).

53. Williams-Guillén, K. The behavioral ecology of mantled howling monkeys (*Alouatta palliata*) living in a Nicaraguan shade coffee plantation. PhD Dissertation, New York University (2003).

54. Behie, A. M. & Pavelka, M. S. M. Food selection in the black howler monkey following habitat disturbance: implications for the importance of mature leaves. *J. Trop. Ecol.* **28**, 153–160 (2012).

55. Felton, A. M. *et al*. Protein content of diets dictates the daily energy intake of a free-ranging primate. *Behav. Ecol.* **20**, 685–690 (2009).

56. Talebi, M. 2005 Factors affecting food choice on the endangered southern muriquis (*Brachyteles arachnoides* - PRIMATE, E. Geoffroy, 1806) in the Brazilian Atlantic forest. PhD Thesis, University of Cambridge (2005).

57. Gaulin, S. J. C. & Craker, L. E. Protein in vegetative and reproductive tissues of several Neotropical species. *J. Agric. Food Chem.* **27**, 791–795 (1979).

58. Castellanos, H. G. & Chanin, P. Seasonal differences in food choice and patch preference of long-haired spider monkeys (*Ateles belebuth*). *Adaptive Radiations of Neotropical Primates* (eds. Norconk, M. A., Rosenberger, A. L. & Garber, P. A.) 451–466 (Plenum Press, 1996).

59. López, M. T., Altrichter, M., Sáenz, J. & Eduarte, E. Valor nutricional de los alimentos de *Tayassu pecari* (Artiodactyla: Tayassuidae) en el Parque Nacional Corcovado, Costa Rica. *Rev. Biol. Trop.* **54**, 687–700 (2006).

60. Beeson, M. Seasonal dietary stress in a forest monkey (*Cercopithecus mitis*). *Oecologia* **78**, 565–570 (1989).

61. Fashing, P. J., Dierenfeld, E. S. & Mowry, C. B. Influence of plant and soil chemistry on food selection, ranging patterns, and biomass of *Colobus guereza* in Kakamega Forest, Kenya. *Int. J. Primatol.* **28**, 673–703 (2007).

62. Agoramoorthy, G. & Hsu, M. J. Borneo's proboscis monkeys–a study of its diet of mineral and phytochemical concentrations. *Curr. Sci.* **89**, 454–457 (2005).

63. Yeager, C. P., Silver, S. C. & Dierenfeld, E. S. Mineral and phytochemical influences on foliage selection by proboscis monkey (*Nasalis larvatus*). *Am. J. Primatol.* **41**, 117–128 (1997).

64. Matsuda, I., Tuuga, A., Bernard, H., Sugau, J. & Hanya, G. Leaf selection by two Bornean colobine monkeys in relation to plant chemistry and abundance. *Sci. Rep.* **3**, 1–6 (2013).

65. Hamilton, R. A. & Galdikas, B. M. F. A preliminary-study of food selection by the orangutan in relation to plant-quality. *Primates* **35**, 255–263 (1994).

66. Waterman, P. G. & Choo, G. M. The effects of digestibility-reducing compounds in leaves on food selection by some Colobinae. *Malays. Appl. Biol.* **10**, 147–162 (1981).

67. Ryan, A. M., Chapman C. A. & Rothman, J. M. How do differences in species and part consumption affect diet nutrient concentrations? A test with red colobus monkeys in Kibale National Park, Uganda. *Afr. J. Ecol.* **51**, 1–10 (2013).

68. Oates, J. F. The distribution of Cercopithecus monkeys in West African forests. *A Primate Radiation: Evolutionary Biology of the African Guenons* (eds. Gautier-Hion, A., Bourlière, F. & Gautier, J.-P.) 79–103 (Cambridge University Press, 1988).

69. Oates, J. F. *et al*. Determinants of variation in tropical forest primate biomass: new evidence from West Africa. *Ecology* **71**, 328–343 (1990).

70. Huang, Z., Sheng, H., Shuguo, Y., Liangwei, C. & Wen, X. Leaf choice by black-and-white snub-nosed monkeys *Rhinopithecus bieti* is related to physical and chemical properties. *Curr. Zool.* **56**, 643–649 (2010).

71. Kar-Gupta, K. & Kumar, A. Leaf chemistry and food selection by common langurs (*Presbytis entellus*) in Rajaji National Park, Uttar Pradesh, India. *Int. J. Primatol.* **15**, 75–93 (1994).

72. Kool, K. M. Food selection by the silver leaf monkey, *Trachypithecus auratus sondaicus*, in relation to plant chemistry. *Oecologia* **90**, 527–533 (1992).

73. Faulkner, A. L. & Lehmann, S. M. Feeding patterns in a small-bodied nocturnal folivore (*Avahi laniger*) and the influence of leaf chemistry: a preliminary study. *Folia Primatol.* **77**, 218–228 (2006).

74. Norscia, I., Ramanamanjato, J. B. & Ganhzorn, J. U. Feeding patterns and dietary profile of nocturnal southern woolly lemurs (*Avahi meridionalis*) in southeast Madagascar. *Int. J. Primatol.* **33**, 150–167 (2012).

75. Donati, G., Bollen, A., Borgognini-Tarli, S. M. & Ganzhorn, J. U. Feeding over the 24-h cycle: dietary flexibility of cathemeral collared lemurs (*Eulemur collaris*). *Behav. Ecol. Sociobiol.* **61**, 1237–1251 (2007).

76. Simmen, B., Bayart, F., Marez, A. & Hladik, A. Diet, nutritional ecology, and birth season of *Eulemur macaco* in an anthropogenic forest in Madagascar. *Int. J. Primatol.* **28**, 1253–1266 (2007).

77. Mutschler, T. Folivory in a small-bodied lemur: the nutrition of the Alaotran gentle lemur (*Hapalemur griseus alaotrensis*). *New Directions in Lemur Studies* (eds. Rakotosamimanana, B., Rasamimanana, H., Ganzhorn, J. U., Goodman, S. M.) 221–239 (Kluwer Academic / Plenum Press, 1999).

78. Eppley, T. M., Verjans, E. & Donati, G. Coping with low-quality diets: a first account of the feeding ecology of the southern gentle lemur, *Hapalemur meridionalis*, in the Mandena littoral forest, southeast Madagascar. *Primates* **52**, 7–13 (2011).

79. Powzyk, J. A. & Mowry, C. B. Dietary and feeding differences between sympatric *Propithecus diadema diadema* and *Indri indri*. *Int. J. Primatol.* **24**, 1143–1162 (2003).

80. Ganzhorn, J. U. *et al*. Selection of food and ranging behaviour in a sexually monomorphic folivorous lemur: *Lepilemur ruficaudatus*. *J. Zool.* **263**, 393–399 (2004).

81. Occhibove, F. et al. Living in islands of forests: nutritional ecology of the howler monkey (*Alouatta palliata*) at La Suerte Biological Field Station, North-eastern Costa Rica. *Central American Biodiversity* (ed. Huettmann, F.) 525–538 (Springer, 2015).

82. Bridgeman, L. L. The feeding ecology of Yucatan black howler monkeys (*Alouatta pigra*) in mangrove forest, Tabasco, Mexico. PhD Thesis, Washington University, 2012.

83. Stevenson, P. R. Fruit choice by woolly monkeys (*Lagothrix lagotricha*) in Tinigua National Park, Columbia. *Int. J. Primatol.* **25**, 367–381 (2004).

84. Mittermeier, R.A., Rylands, A.B. & Wilson D.E. *Handbook of the Mammals of the World: Volume 3 Primates* (Lynx Edicions, 2013).
